# Comprehensive review of Wolbachia research (1936–2024): Global landscape, mapping progress and themes

**DOI:** 10.1016/j.parepi.2025.e00438

**Published:** 2025-06-10

**Authors:** Manal Mohamed Elhassan Taha, Siddig Ibrahim Abdelwahab, Hafeez Yagoub Mohamed, Ahmed Jerah, Aied M. Alabsi, Saleh Mohammad Abdullah, Bassem Oraibi, Hassan Ahmad Alfaifi, Yasir Osman Hassan Babiker, Ibrahim Abdel Aziz Ibrahim, Saeed Alshahrani, Abdullah Mohammed Farasani, Ahmed S. Alamer, Tawfeeq Altherwi

**Affiliations:** a Health Research Centre, Jazan University, Jazan, Saudi Arabia; b College of Medicine, Najran University, Najran, Saudi Arabia; c Department of Medical Laboratory Technology, College of Nursing and Health Sciences, Jazan University, Jazan, Saudi Arabia; d Management and Science University, University Drive, Off Persiaran Olahraga, 40100 Shah Alam, Selangor, Malaysia; e Pharmaceutical Care Administration (Jeddah Second Health Cluster), Ministry of Health, Jeddah, Saudi Arabia; f Department of Surgery, College of Medicine, Jazan University, Jazan, 45142, Saudi Arabia; g Department of Pharmacology and Toxicology, Faculty of Medicine, Umm Al-Qura University, Makkah, Saudi Arabia; h Department of Pharmacology and Toxicology, College of Pharmacy, Jazan University, Saudi Arabia; i Department of Health Education and Promotion, Faculty of Public Health and Tropical Medicine, Jazan University, Jazan, Saudi Arabia; j Department of Internal Medicine, Faculty of Medicine, Jazan University, Jazan, Saudi Arabia

**Keywords:** *Aedes aegypti*, Bibliometrix, *Wolbachia*, wMel, wAlbB, VOSviewer

## Abstract

*Wolbachia*, an obligatory gram-negative intracellular bacterium associated with *Rickettsia*, was initially identified in *Culex pipiens* mosquitoes and later in diverse invertebrates. This study utilizes bibliometric methodologies to quantitatively analyze *Wolbachia* research (WR), filling a gap in systematic analysis. Following PRISMA guidelines, original English papers were extracted from Scopus and analyzed using VOSViewer and Bibliometrix to assess performance indices, citations, co-word mapping, emerging themes, and the evolution of WR. Since its inception between 1936 and 1961, WR has grown to 4800 documents by 2024, with notable surges in 2022 and 2024. Scholars like O'Neill, Hoffmann, and Bourtzis have significantly influenced this field. Bradford's law highlights WR distribution among 876 sources, with 37.54 % of studies being collaborative. Six thematic areas have evolved toward practical applications, particularly in vector control and disease management. Emerging topics since 2015, such as “cytoplasmic incompatibility” and “arboviruses,” reflect growing interest in microbiology and disease control.

## List of abbreviations

**Table:** 

*Ae. aegypti*	*Aedes aegypti*
CHIKV	Chikungunya Virus
CI	Cytoplasmic Incompatibility
CNRS	Centre National de la Recherche Scientifique (French National Centre for Scientific Research)
DENV	Dengue Virus
G-index	Egghe's G-index
H-index	Hirsch index
mtDNA	Mitochondrial DNA
NP	Number of Papers
PY_start	Year of First Publication
RNA	Ribonucleic Acid
Scopus	An abstract and citation database
SFV	Semliki Forest Virus
TC	Total Citations
TLS	Total Link Strength
UK	United Kingdom
USA	United States of America
VOSviewer	Visualization of Similarity Viewer
WR	*Wolbachia* Research
YFV	Yellow Fever Virus
ZIKV	Zika Virus

## 1 Introduction

*Wolbachia* is an gram-negative bacterial endosymbiont that infects a significant proportion of arthropod species and filarial nematodes worldwide (Porter and Sullivan, 2023). Effective vertical and horizontal transmission, modification of host reproduction, and augmentation of host fitness can facilitate dissemination both within and beyond species. *Wolbachia* is prevalent and can inhabit a remarkably varied range of evolutionarily distant host species, indicating that they have evolved to interact with and regulate highly conserved fundamental cellular processes (Hoffmann et al., 2011; Hoffmann et al., 1990; Nazni et al., 2019; Turelli and Hoffmann, 1995).

*Wolbachia is a common intracellular symbiotic bacterium that is maternally inherited in many insect species (*Weeks et al., *2007**)*. One of its most notable effects is cytoplasmic incompatibility (CI), a form of reproductive manipulation in which sperm from Wolbachia-infected males is modified in a way that results in embryo death when they mate with uninfected females. This mechanism gives a reproductive advantage to infected females, promoting the spread of Wolbachia through insect populations (Hoffmann et al., 2011; Nazni et al., 2019; Hoffmann et al., 2014; Walker et al., 2011; Zheng et al., 2019). In addition to its role in reproductive biology, Wolbachia has gained attention for its ability to block the transmission of several human pathogens. For instance, *Aedes aegypti* mosquitoes—primary vectors for dengue (DENV), Zika (ZIKV), yellow fever (YFV), and chikungunya (CHIKV)—do not naturally carry Wolbachia, but when artificially infected, these mosquitoes show a significantly reduced ability to transmit these flaviviruses, which pose major public health threats across tropical region (Utarini et al., 2021; Tan et al., 2017).

Numerous investigations have demonstrated that a key factor influencing the relative capacity of different *Wolbachia* strains to suppress viruses is their intracellular density (Nazni et al., 2019; Hoffmann et al., 1998). However, recent evidence has suggested that density is not the only factor at play (Lu et al., 2012). For example, high intracellular densities were achieved when wAu and wAlbA strains, which originated in *Drosophila simulans* and *A. albopictus*, respectively, were transferred to *Ae. aegypti (*Nazni et al., *2019**;* Utarini et al., *2021**;* Tan et al., *2017**)*. However, wAlbA demonstrated limited antiviral activity against DENV/SFV and a relatively weak capacity to inhibit ZIKV *in vivo*. In contrast, wAu effectively inhibited virus transmission, and there were no signs of DENV spreading past the midgut. Lipid transport and metabolism have been shown to play mechanistic roles in the capacity of wMel/wMelPop strains (derived from *Drosophila melanogaster*) to suppress DENV *in vivo* and *in vitro* in *Ae. aegypti*. In the wMel/wMelPop-carrying *Ae. aegypti* cells, there is an increase in the sequestration of cholesterol into lipid droplets (Chouin-Carneiro et al., 2020; Martinez et al., 2022). Treatment with cyclodextrin 2HPCD released stored cholesterol and caused partial recovery of DENV replication. However, these modifications have been applied to all *Wolbachia* strains that suppress viruses (Geoghegan et al., 2017).

Release projects employing strains wMel or wAlbB, which originate from *A. albopictus*, are being conducted in several countries to restrict the spread of DENV (Zheng et al., 2019; Chouin-Carneiro et al., 2020; Martinez et al., 2022). These strains carry *Wolbachia*. In Malaysia, a wAlbB-based intervention experiment demonstrated a 40–80 % decrease in dengue incidence across several release sites (Liang, 2020). It is becoming increasingly crucial to understand the molecular mechanisms underlying *Wolbachia*-mediated antiviral activity owing to the organism's ongoing field deployment. Understanding the processes of viral suppression may help in the monitoring and mitigation of possible operational issues, such as the potential for viral “escape” mutations or instability of specific symbiont strains under specific conditions. Raising *Ae. aegypti* larvae at temperatures above approximately 35 °C reduces both the density and maternal transmission of wMel, which may compromise their ability to block DENV in hot environments and increase the possibility of escape mutation selection (Nazni et al., 2019; Hoffmann et al., 1998; Lu et al., 2012; Chouin-Carneiro et al., 2020; Martinez et al., 2022). The long-term success of the strategy would be greatly enhanced if *Wolbachia* strains with viral inhibition that differs mechanistically from wMel/wAlbB could be found for use in release programs. This would enable a way to mitigate the risk of viral escape, should it materialize, or lessen the likelihood that mutations leading to viral escape will be selected (Hoffmann et al., 2011; Nazni et al., 2019; Hoffmann et al., 2014; Chouin-Carneiro et al., 2020; Martinez et al., 2022; Geoghegan et al., 2017; Liang, 2020).

Bibliometric research was originated in the late 19th century. Bibliometrics involves the utilization of statistical techniques to analyze bibliographic data, particularly within scientific, library, and information science domains, and is strongly linked to scientometrics, resulting in a significant overlap between the two subjects. Numerous programs have facilitated bibliometric analyses. The Bibliometrix R Package and its web application, Biblioshiny, along with VOSviewer and CiteSpace, are frequently used software tools for bibliometric data analysis (Aria and Cuccurullo, 2017; Pritish Baskaran et al., 2023; Tsai et al., 2020; Tseng et al., 2020; Van Eck and Waltman, 2010). Notwithstanding the significance of *Wolbachia* research (WR) in disease vector management, no prior bibliometric analyses have examined worldwide output, contemporary and prospective trends, or conceptual evolution. Therefore, the current study was designed to analyze research related to *Wolbachia* from 1936 to 2024. This bibliometric analysis, covering over eight decades, serves scholars and research-funding organizations.

## 2 Materials and methods

### 2.1 Source of bibliographic data and search strategy

The current study employed the Scopus database to extract data related to WR. Scopus, developed by Elsevier, is an abstract and indexing database, complete with full-text links. The name Scopus is derived from Hammerkop (*Scopus umbretta*), a bird known for its exceptional navigational ability. The database, developed for two years, was created in collaboration with 21 academic institutions and over 300 researchers and librarians. Feedback from these librarians and researchers was examined and utilized to enhance the product. Scopus creators assert that they index more than 14,000 STM (Science, Technology, and Medicine) and social scientific titles from 4000 publishers, claiming it to be the “largest single abstract and indexing database ever constructed.” The database asserts that it indexes 4600 health science titles encompassing complete MEDLINE, EMBASE, and Compendex coverage (Burnham, 2006; Kulkarni et al., 2009). The term “*Wolbachia*’ was used to generate the initial data from ABS-TITLE-KEYWORDS in Scopus. The search query for “*Wolbachia*” in Scopus yielded 4893 articles, 524 reviews, 148 book chapters, 100 notes, 85 conference papers, 71 short surveys, 58 letters, 54 editorials, 43 errata, eight books, three conference reviews, and three data papers. The data for this study was extracted in on June 1, 2024.

### 2.2 Inclusion and exclusion criteria

**Fig. 1:**
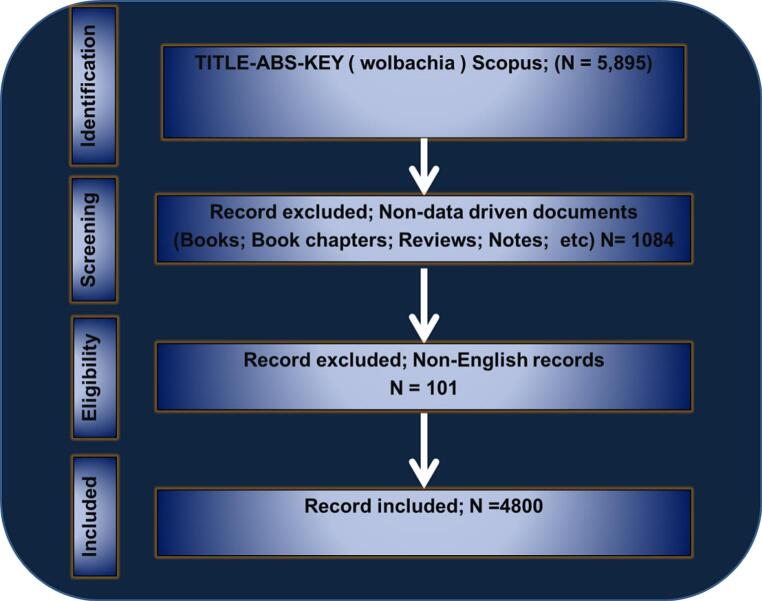
Data selection and extraction according to the PRISMA guidelines.

The PRISMA principles (Sarkis-Onofre et al., 2021) were adhered to when assessing the eligibility of publications for inclusion (Fig. 1). The criteria encompassed studies published in English, data-driven original articles, articles indexed in Scopus, and articles with no temporal restrictions. The total number of papers covered in this study was 4800.

### 2.3 Data analysis and visualization

VOSviewer (1.16.20) and Bibliometrix (4.3.1) software were used for data analysis (Aria and Cuccurullo, 2017; Van Eck and Waltman, 2010). These tools were used to analyze the bibliometric data; identify the leading authors, institutions, and areas of research; and create graphical representations of the research domain. Different evaluation criteria were applied in the research: the temporal structure of output, number of authors, title of papers, volume of collaborating authors and countries, rates of citations, occurrence of keywords, and localization of the research themes. Other metrics such as the H-index, G-index, and M-index were used to evaluate author performance. The H-index reflects both productivity and impact by measuring the number of publications (h) that have received at least h citations, offering an indication of consistent scholarly contribution. The G-index gives more weight to highly cited papers, emphasizing the influence of publishing in high-impact journals. The M-index, defined as the H-index divided by the number of years since the researcher's first publication, helps normalize performance over time and highlights sustained productivity. In this study, these metrics were applied to assess the degree of individual contribution to collaborative authorship. Together, they offer a balanced view of both the influence and output volume of authors in Wolbachia research (Manjareeka, 2023).

Bradford's law in bibliometric analysis describes how scholarly articles are distributed across journals in a field and identifies a small number of core journals that publish the majority of relevant research. The law divides journals into three zones: core journals with the most articles, and two progressively larger zones with fewer articles per journal. The number of journals in each zone increased exponentially. This principle helps researchers and librarians focus on the most productive sources in a discipline, aiding in journal selection and optimizing research access (Aria and Cuccurullo, 2017). This review has been carried out thematically and seeks to arrange the theme-map analysis results into a narrative review. The thematic map can be seen as a diagram on which primary themes and sub-themes of a particular field of study are sketched to aid scholars to further their studies in the literature and relate how the various fields of research interconnect. This study developed a thematic map based on co-word network analysis and clustering (Aria and Cuccurullo, 2017; Afuye et al., 2021; Alkhammash, 2023; Bérdi, 2023). The analysis identified clusters of related keywords by analyzing the occurrence patterns of phrases in the literature, thus illuminating the thematic organization of WR. Similar to the above assertion, this approach enables a more descriptive element to be added to the study of the research domain.

## 3 Results

### 3.1 Inception, growth and impact of WR

**Fig. 2:**
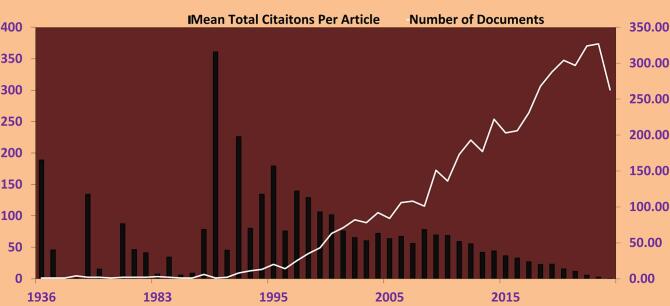
From 1936 to 2024, *Wolbachia* research (WR) shows 4800 papers published overall with an average document age of 9.61. Research activity has lately surged especially between 2022 and 2024. Each document has an average citation count of 38.15; some years, like 1990, show clear spikes in this count.

Based on Fig. 2, the field of Wolbachia research (WR) has shown substantial progress between 1936 and 2024. The earliest publication was in 1936, titled “The *Rickettsia*, *Wolbachia pipientis* (Gen. et Sp. N.) and Associated Inclusions of the Mosquito, *Culex pipiens*,” followed by a 1961 study “Isolation of a *Rickettsia*like Microorganism (*Wolbachia persica*, n. sp.) from *Argas persicus* (Oken).” Over the years, a total of 4800 documents related to WR have been published, with a mean publication age of over nine years. Notably, publication activity has intensified in recent years, with 324 documents published in 2022 and 263 in 2024, reflecting growing interest in the field. The past decade (2014–2024) recorded the highest output, with 2409 documents—accounting for more than 50.19 % of all WR publications—indicating that research activity more than doubled during this period.

Fig. 2 illustrates how WR has evolved over time. Compared to other fields, WR authors demonstrate a high level of scholarly engagement, with an average of 38.15 citations per document. Foundational studies from 1936 and 1961 received significant attention, with a combined 189 citations, which likely influenced subsequent research directions. Citation peaks, including those in 1990 and beyond, highlight pivotal moments in WR development. However, the average number of citations per article has declined, from 44.34 in 2014 to 0.46 in 2024, possibly indicating a shift in research focus or saturation in citing earlier seminal works. These citation trends help trace the evolution of WR and signal shifts in influence and thematic interest over time.

### 3.2 Influential scholars

The bibliometric study of eminent academics in WR reveals notable contributions from key figures (Table 1). With 14,089 authors contributing to WR, the field demonstrates a broad and diverse range of perspectives and expertise. O'Neill, S.L. led the field with an H-index of 66, a G-index of 133, and a total of 17,509 citations across 144 publications, marking him as the most cited and prolific researcher since 1989. Hoffmann, A.A., with a notable H-index of 49 and 138 publications, also played a crucial role, amassing 9668 citations since the same year. Werren, J.H. and Bourtzis, K. followed closely, with Werren, achieving 11,372 citations and a strong M-index of 1.484, indicating the consistent impact. Researchers such as Taylor, M.J., Hoerauf A., and Bordenstein, S.R. have made significant strides in the field, contributing between 62 and 80 publications each.

**Table 1:** Most impactful scholars.

Element	H_index	G_index	M_index	TC	NP	PY_start
O'neill, S.l.	66	133	1.833	17,809	144	1989
Hoffmann, A.A.	49	97	1.361	9668	138	1989
Werren, J.H.	46	73	1.484	11,372	73	1994
Bourtzis, K.	38	71	1.226	5274	71	1994
Taylor, M.J.	38	66	1.462	4400	73	1999
Hoerauf, A.	37	70	1.423	4938	80	1999
Bordenstein, S.R.	36	62	1.333	5791	62	1998
Hurst, G.D.D.	35	60	1.25	4594	60	1997
Mcgraw, E.A.	32	62	1.333	6633	62	2001

TC: total citations; NP: number of papers; PY_start: first year of publication.

### 3.3 Bradford's law

**Fig. 3:**
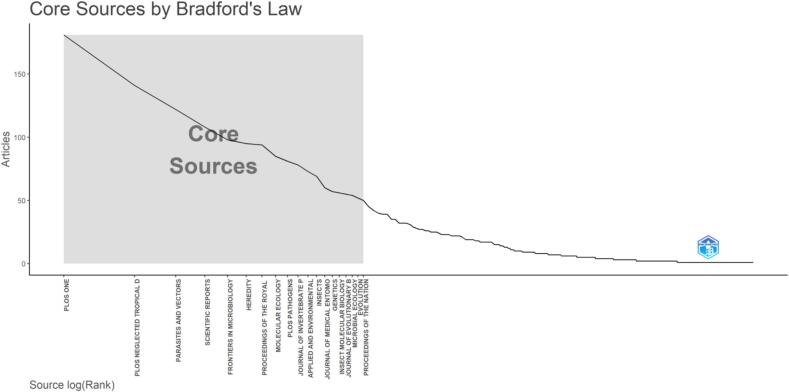
Bradford's law for the determination of the top sources in *Wolbachia* research. Notable sources in this subject, such PLOS ONE, PLOS NEGLECTED TROPICAL DISEASES, and SCCIENTIFIC REPORTS, occupy Zone 1 according to the law, thereby showing their importance as abundant sources of *Wolbachia*-related studies. This figure was generated using the Bibliometrix application.

Bradford's law in bibliometric analysis describes how scholarly articles are distributed across journals in a field, identifying a small number of core journals that publish the majority of relevant research (Aria and Cuccurullo, 2017). In WR, Bradford's law illuminates the distribution of scholarly articles across journals (*N* = 876), pinpointing a select group of core journals that serve as primary disseminators of pivotal research findings (Fig. 3). Notable sources in WR, such as PLOS ONE, PLOS NEGLECTED TROPICAL DISEASES, and SCCIENTIFIC REPORTS, occupy Zone 1 according to law, thereby showing their importance as abundant sources of *Wolbachia*-related studies. Within this central zone, journals such as PARASITES, VECTORS, FRONTIERS IN MICROBIOLOGY, and HEREDITY highlight their involvement in forming the scientific debate around *Wolbachia*. By concentrating on these fundamental publications, librarians and researchers can effectively negotiate the vast terrain of *Wolbachia* literature, thereby guaranteeing access to important research insights and supporting informed decisions on journal choice for the best research outcomes in this particular subject.

### 3.4 Prolific affiliations and countries

**Fig. 4:**
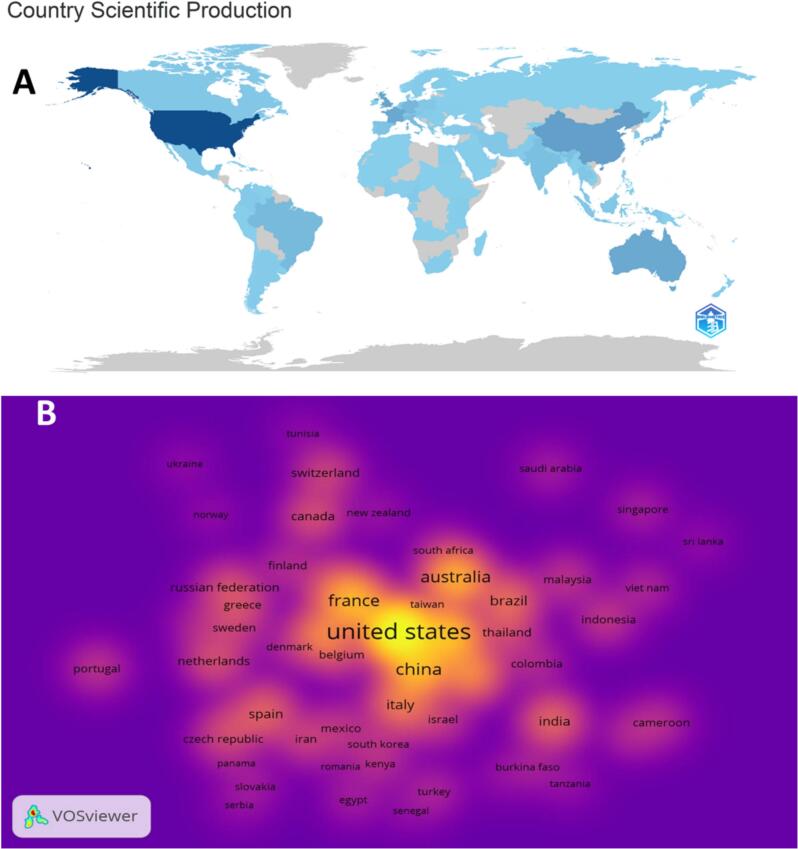
A: Global landscape of *Wolbachia* research. The intensity of the blue color in Fig. 4A represents the number of published documents. B: Highly cited countries. The intensity of yellow color in Fig. 4B represents the number of citations. (For interpretation of the references to color in this figure legend, the reader is referred to the web version of this article.)

Among the leading affiliations contributing to WR, the data highlights institutions including Centre National de la Recherche Scientifique (France), The University of Queensland, University of Melbourne (Australia), Liverpool School of Tropical Medicine (UK), Monash University (Australia), Bio21 Molecular Science and Biotechnology Institute (Australia), Université de Montpellier (France), University of Rochester (USA), and Université de Poitiers (France) as most prolific in this field. These institutions have played a significant role in research that advances our understanding of *Wolbachia* and its interactions with other organisms. The most prolific countries (Fig. 4A) in WR include the USA (1592), the UK (638), China (568), France (546), Australia (517), Germany (312), Japan (289), Italy (234), and Brazil (205). The intensity of the blue color in Fig. 4A represents the number of published documents. In WR, the USA was the most cited country (Fig. 4B) with 86,992 citations, followed by the UK (35309), Australia (29398), France (23218), Germany (14653), China (13255), Japan (12548), Italy (9451), and Brazil (8077). These countries have significantly influenced the field through their research, contributing to the scholarly impact and advancement of knowledge in *Wolbachia* and its implications across diverse scientific disciplines. The intensity of the yellow color in Fig. 4B represents the number of citations.

### 3.5 Mapping of co-authorship

**Fig. 5:**
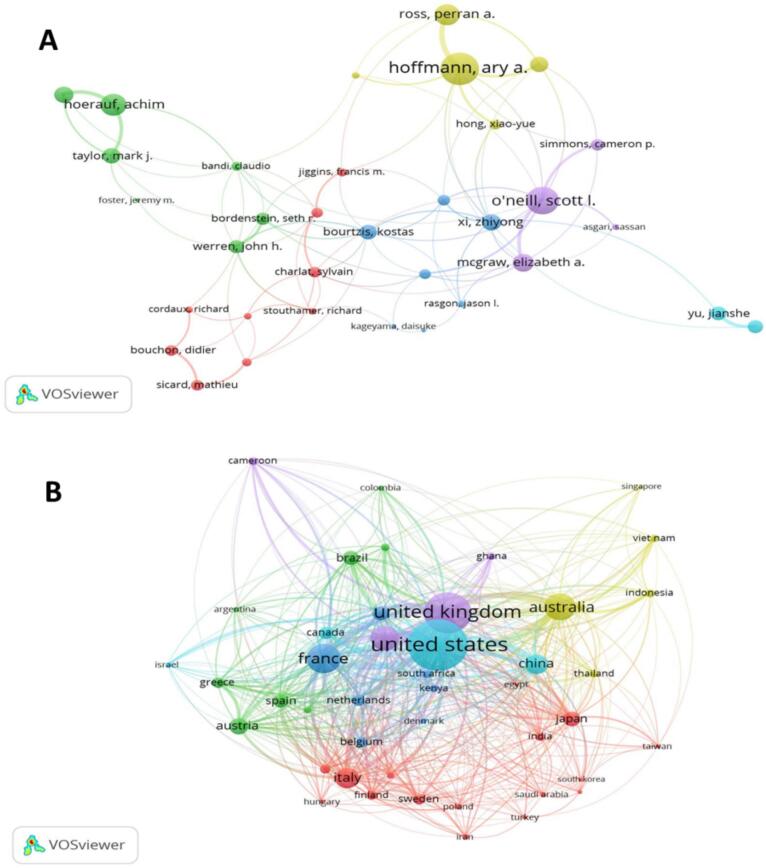
In VOSviewer, Total Link Strength (TLS) represents the overall strength of connections between nodes (*e.g.*, authors, institutions). In co-authorship analysis, it quantifies the intensity of collaboration between authors (4A) or countries (4B), indicating stronger partnerships in WR. The size of the nodes represents the corresponding TLS value of each author or country.

In WR, collaboration is integral, with the vast majority of documents having multiple authors (an average of 5.8 co-authors per document). Only 154 documents were single-authored, emphasizing the collective effort to advance knowledge in this field. International co-authorships represent 37.54 % of collaborations, highlighting a global network of researchers pooling diverse perspectives to explore *Wolbachia* biology and applications. In VOSviewer, Total Link Strength (TLS) represents the overall strength of the connections between nodes (*e.g.*, authors and countries). The co-authorship analysis quantifies the intensity of collaboration between authors or institutions, indicating stronger partnerships in WR (Fig. 5A and B). Among the authors (Fig. 5A), Hoffmann, A. A. (79) has the highest TLS, reflecting extensive collaboration networks, with O'Neill, S. L. (63), Hoerauf, A. (45), Ross, P. A. (43), and McGraw, E. A. (34) also playing key roles. In WR, the co-authorship analysis shows that the United States (1271) leads to TLS, indicating the strongest international collaborations, followed by the United Kingdom (907), France (572), Australia (514), and Germany (439) (Fig. 5B). These metrics highlight the most influential countries and authors in shaping global research collaboration in WR.

### 3.6 Diffusion of knowledge

**Fig. 6:**
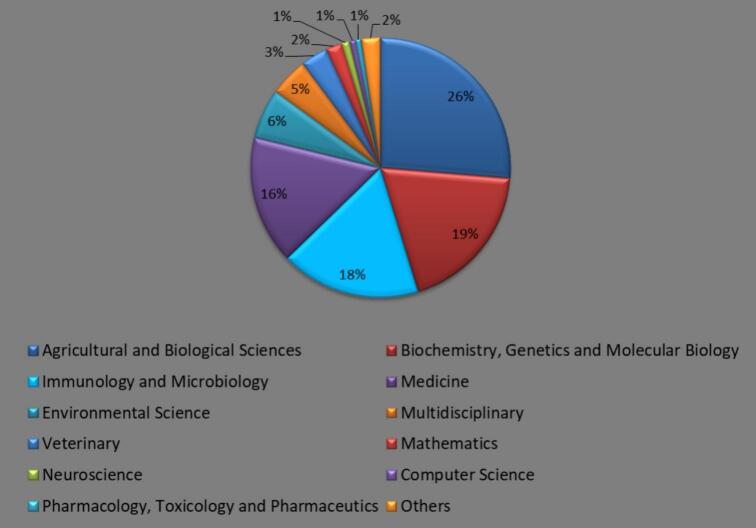
Diffusion of *Wolbachia* related knowledge across the various fields of research according to the Scopus classification of subjects. Others subjects (2 %), include Pharmacology, Toxicology and Pharmaceutics, Chemistry, Engineering, Physics and Astronomy, Chemical Engineering, Earth and Planetary Sciences, Social Sciences, Materials Science, Economics, Econometrics and Finance, Health Professions, Business, Management and Accounting, Decision Sciences, Nursing, Arts and Humanities and Psychology.

The diffusion of WR spans various fields based on Scopus criteria (Fig. 6). Agricultural and Biological Sciences accounted for 26.32 % of the research, followed by Biochemistry, Genetics, and Molecular Biology at 18.95 %, Immunology and Microbiology at 17.48 %, and Medicine at 16.03 %. Environmental Science contributes 6.22 %, while Multidisciplinary fields cover 4.78 %. Other areas include Veterinary (3.39 %), Mathematics (2.04 %), Neuroscience (0.98 %), Computer Science (0.79 %), Pharmacology, Toxicology, and Pharmaceutics (0.68 %), Chemistry (0.66 %), Engineering (0.38 %), Physics and Astronomy (0.35 %), Chemical Engineering (0.34 %), Earth and Planetary Sciences (0.17 %), Social Sciences (0.15 %), Materials Science (0.09 %), Economics, Econometrics, and Finance (0.06 %), Health Professions (0.05 %), Business, Management, and Accounting (0.03 %), Decision Sciences (0.02 %), Nursing (0.02 %), Arts and Humanities (0.01 %), and Psychology (0.01 %).

### 3.7 Co-word analysis and conceptual mapping

**Fig. 7:**
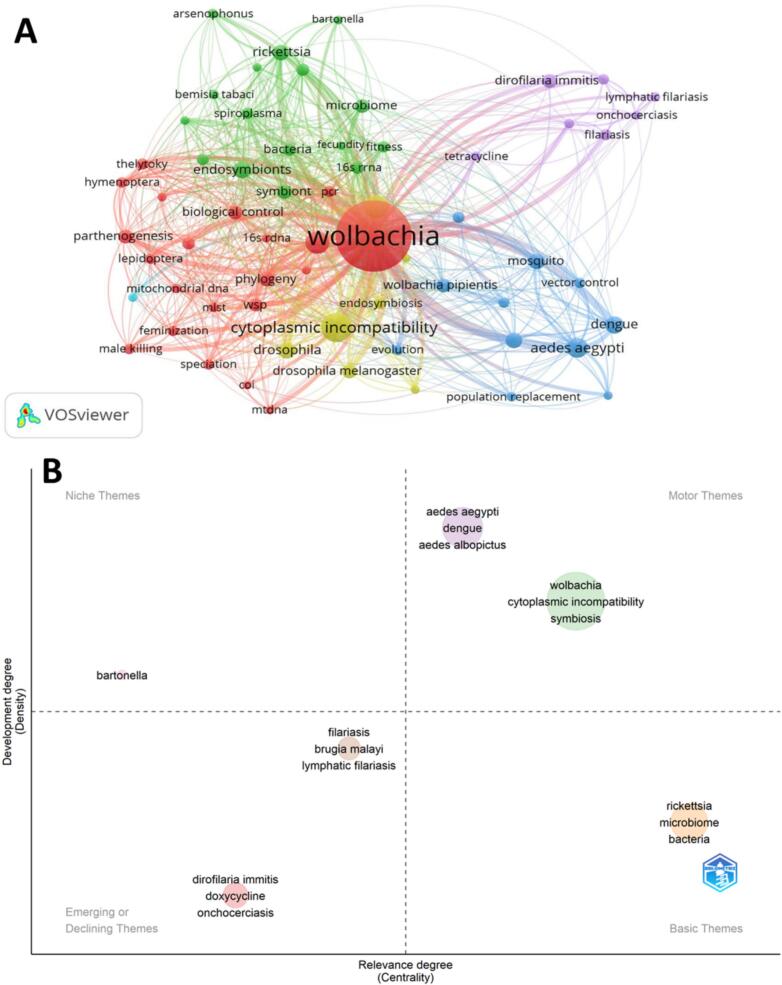
Co-word analysis. A: Most frequent author's keywords. VOSviewer was used to extract the most frequent keywords. Out 6966 keywords, 63 with minimum frequencies were mapped. B: Thematic mapping was drawn using Bibliometrix application. The map is divided into four quadrants based on the centrality and density of the themes.

A co-word analysis examines the co-occurrence of author keywords to identify patterns and emerging themes within a field. Conceptual mapping visualizes these relationships and creates networks of related terms to highlight a field's core topics and trends. Together, they provided insights into the conceptual structure of the research domain. By analyzing the frequency and proximity of keywords, co-word analysis helps reveal the conceptual structure of a research domain and identify key topics and their interconnections (Tsai et al., 2020; Tseng et al., 2020; Afuye et al., 2021; Alkhammash, 2023). The most frequent author's keywords in the WR (Fig. 7A) include terms such as “*Wolbachia*” (1961 occurrences), “cytoplasmic incompatibility” (327), “symbiosis” (208), “endosymbiont” (201), “*Ae. aegypti*” (158), “Dengue” (115), “endosymbionts” (109), “*Drosophila*” (106), “*A. albopictus*” (98), “*Rickettsia*” (98), “mosquito” (94), “phylogeny” (85), “*Drosophila melanogaster*” (77), “parthenogenesis” (76), “biological control” (74), “microbiome” (74) and “*Dirofilaria immitis*” (74) among others (Fig. 7A). The thematic map of WR encompasses six key themes (Fig. 7B) classified as follows: “*Wolbachia*” and “*Ae. aegypti*” are identified as motor themes driving research in the field, while “*Rickettsia*” represents a basic theme fundamental to understanding. “*D. immitis*” is recognized as an emerging theme indicating growing interest and relevance, whereas “*Bartonella*” is categorized as a niche theme focusing on specific areas. Conversely, “*Filariasis*” was classified as a declining theme, suggesting a decreasing emphasis or interest in this particular aspect of the WR landscape.

### 3.8 Thematic dynamicity

**Fig. 8:**
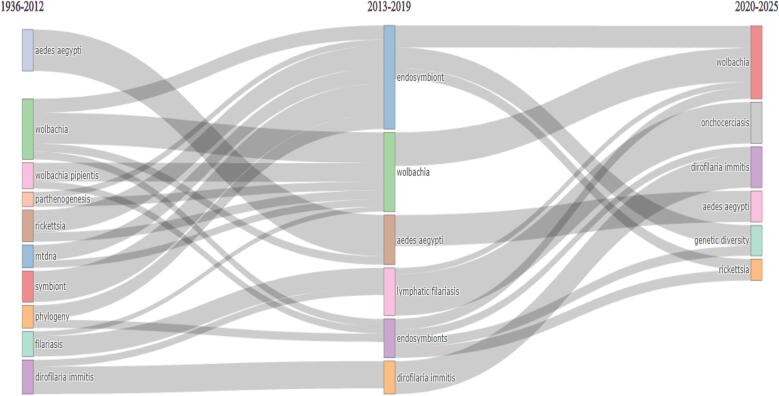
The thematic evolution of Wolbachia research since 1936 till 2025.

Fig. 8 presents the thematic evolution of WR across the three periods: 1936–2012, 2013–2019, and 2020–2025. This graph shows the importance of the study subjects, and *Wolbachia*-related terminology—*Ae. aegypti*, *D. immitis*, endosymbionts, and filariasis— has changed in focus over time. From 1936 to 2012, the fundamental subjects, including *Ae. aegypti*, mtDNA, parthenogenesis, and symbionts, took the front stage. From 2013 to 2019, these subjects were changed to highlight endosymbionts, lymphatic filariasis, and more studies on *Wolbachia* and its use. Research has progressed toward more specialized subjects, including genetic diversity, onchocerciasm, and ongoing attention to *Wolbachia* and its function in regulating diseases carried by *Ae. aegypti* in the latest period (2020–2025). Particularly in vector control and disease management, this table shows the changing objectives and patterns within WR, thereby demonstrating the shift from fundamental biological elements to more practical research.

### 3.9 Evolving WR

**Fig. 9:**
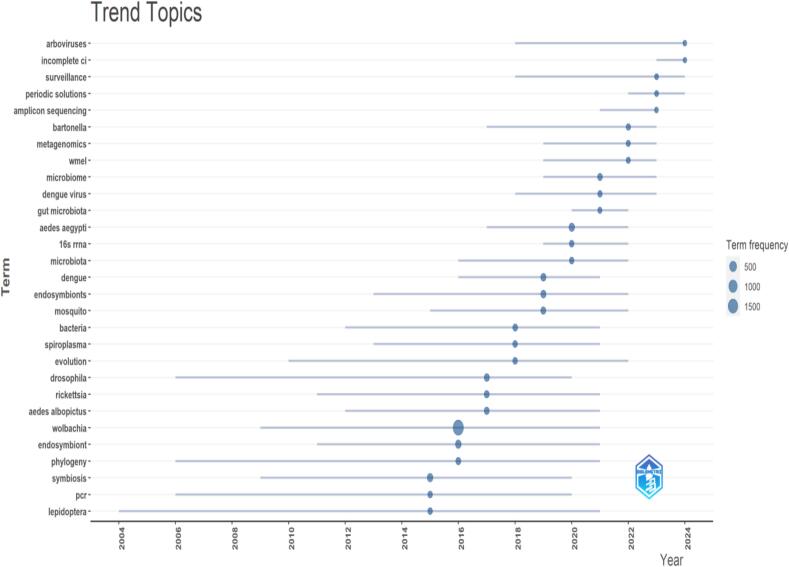
The evolving topics in *Wolbachia* research during the last two decades. Lines denote the lifespan of the topic, whilst circles indicate the density of the topic.

Since 2015, “cytoplasmic incompatibility,” “periodic solutions,” “amplicon sequencing,” “gut microbiota,” “16S rRNA,” “microbiota,” “metagenomics,” “wMel,” “Dengue virus,” “surveillance,” “arboviruses,” “*Ae. aegypti*,” “*Bartonella*,” “Dengue,” “microbiota,” and “mosquito,” have been the emerging subjects in research. These new themes (Fig. 9) underline the changing terrain of research in these disciplines since 2015 by highlighting the increasing interest and focus within the scientific community in many aspects, including microbiology, genomics, disease surveillance, vector biology, and microbiota dynamics related to arboviruses and mosquito-borne diseases such as dengue.

## 4 Discussion

This study aimed to provide a complete bibliometric analysis of WR, between the years 1936–2024 encompassing global publication output, trends, and conceptual development. The main advantage of this research is the time span and utilization of modern bibliometric instruments such as Bibliometrix and VOSviewer, which deepens our understanding of publication activity and new research directions. This study is unique in that it is the first attempt to perform a bibliometric analysis of WR, which has not been studied despite its relevance in vector-borne disease control.

Several factors accounted for the considerable expansion of the WR after the 1990s. In this regard, it is especially worth noting the change from merely studying biological clients to using biological techniques to combat diseases transmitted by mosquitoes, such as Dengue, Zika, and malaria. Fundamental studies initiated in 1936 (Hertig, 1936) and 1961 (Suitor and Weiss, 1961) were in place; however, WR took off when *Wolbachia* was found to reduce viral loads or cause CI in mosquitoes (Hoffmann et al., 1990; Turelli and Hoffmann, 1995; Hoffmann et al., 1996). This brought the focus back to innovations in the 1990s and the peak mean number of citations (361 in 1990) for biological control strategies during research. Except for the healthy increase in output from 2014 to 2024, in which WR output accounted for 50.19 % of total publications, the grounds for improved WR performance are likely to be international projects and field trials with mosquitoes carrying *Wolbachia*. However, the decrease in citations per article from 44.34 in 2014 to 0.46 in 2024 suggests a delay in the most recent publications. This implies that this field might be developing toward either discovering new or bettering current methods. This evolution underlines WR's growing relevance in addressing public health issues. The findings of a prior bibliometric analysis of mathematical modeling studies on dengue management with *Wolbachia* bacteria (Hasibuan et al., 2022) align with the outcomes of the present study. Moreover, various bibliometric analyses of the dengue virus have implicitly shown an increase in research pertaining to *Wolbachia* (Liu et al., 2024; Ho et al., 2016; Halim et al., 2021).

O'Neill, S. L. made major contributions to WR while working at Monash University's Institute of Vector-Borne Disease in Australia. His research has explored the complex interactions between Wolbachia and arboviruses such as chikungunya and yellow fever viruses in the mosquito vector Ae. aegypti, shedding light on how Wolbachia influences infection dynamics. O'Neill and colleagues also investigated the potential of Wolbachia symbionts to prevent the transmission of pathogens such as Plasmodium, chikungunya, and dengue viruses within mosquito populations. A significant focus of his work has been the stable introduction of Wolbachia into Ae. aegypti mosquitoes, offering innovative strategies for vector control. Overall, O'Neill's work has been instrumental in shaping current understanding of Wolbachia's role in mosquito biology and its application in disease transmission control (van den Hurk et al., 2012; Moreira et al., 2009; McMeniman et al., 2009; Hedges et al., 2008; Wu et al., 2004; Dobson et al., 1999; Braig et al., 1998; O'Neill et al., 1992).

Attached to the Bio21 Institute and the Department of Genetics at the University of Melbourne, Hoffmann, A.A. has made pioneering contributions to WR, especially in terms of knowledge of its function in population dynamics and its application in the management of mosquito-borne diseases, such as dengue. His studies on naturally occurring *Wolbachia* infections in *Drosophila melanogaster* and *Drosophila simulans* have shed important light on CI, a mechanism that allows *Wolbachia* to proliferate in insect populations. Hoffmann expanded this study to *Ae. aegypti*, the main dengue vector, in which he examined the stability and establishment of *Wolbachia* strains, such as wMel and wAlbB, thereby proving their capacity to stop dengue transmission and preserve their presence in mosquito populations. His investigations with a Malaysian team and other affected areas have revealed the effective field establishment of these strains, thereby greatly supporting dengue control initiatives. His work provides new, ecologically acceptable methods to fight vector-borne diseases, thereby bridging the gap between evolutionary biology and public health (Hoffmann et al., 2011; Hoffmann et al., 1990; Nazni et al., 2019; Turelli and Hoffmann, 1995; Weeks et al., 2007; Hoffmann et al., 2014; Walker et al., 2011; Zheng et al., 2019; Hoffmann et al., 1998; Hoffmann et al., 1996).

This section presents information on the publication output in WR of major contributors such as CNRS, the University of Queensland, and Monash University, who have deepened their knowledge of the properties of *Wolbachia* in the fight against diseases. The USA shows the highest output (1592 papers) and number of citations (86992), with the next places occupied by countries like the UK, Australia, China that impacted this area greatly. There is a need for bibliometric mapping of collaboration because it is equally important, as it pinpoints these global bonds helping in knowledge exchange and making the research networks stronger. This international cooperation also proves that WR and its application in healthcare, including disease prevention, genetics, and vector-borne diseases, are of great importance. Two bibliometric studies also corroborate the cross-country collaboration features in vector control, which is in agreement with our findings on *Wolbachia* studies. For instance, Noden (2016) emphasized the need for international collaborations to make consistent progress in vector-borne disease control (Noden, 2016) whereas Zyoud (2016) stressed that dengue action requires more collaborative conduct (Zyoud, 2016). Such studies exemplify the great involvement of nations, including the United States, the United Kingdom, and Australia, in these studies, simply regarding the worldwide resolution of vector-borne disease control is more plausible.

The Scopus criteria (Fig. 6) show the transdisciplinary nature of WR. Agricultural and Biological Sciences dominated the research landscape at 26.32 %, demonstrating *Wolbachia*'s impact on agriculture, pest management, and biological systems (Hoffmann et al., 2011; Nazni et al., 2019; Walker et al., 2011; Zheng et al., 2019; Bérdi, 2023). Following closely, Biochemistry, Genetics, and Molecular Biology (18.95 %) emphasize *Wolbachia* interactions' genetic and molecular complexity (Nazni et al., 2019; Martinez et al., 2022). calculated figures attributable to Immunology and Microbiology (17.48 %) appreciate *Wolbachia*'s role in microbial ecosystems. In Medicine, the prevalence of the *Wolbachia* is approximately 16.03 %, and its domains bring about control of diseases and treatments. As Environmental Science (6.22 %) indicates, there are also impacts of *Wolbachia* applications on the environment and the ecology. It requires research across disciplines and contains 4.78 % of multidisciplinary domains. There are also *Wolbachia* applications in Veterinary Science (3.39 %) and Mathematical Science (2.04 %) (Adekunle et al., 2019; Zhang and Lui, 2020; Andreychuk and Yakob, 2022; Yang et al., 2023). WR characteristics in Neuroscience, Computer science, and chemistry reveal transdisciplinary expansion trends (Guevara-Souza and Vallejo, 2015). Studies in Engineering, physics, Earth, Planetary Sciences, and Social Sciences have advanced the application of *Wolbachia* study to new fields, and thus its cross-cutting and dimensional characteristics. That is, Health Professions Drive activities to minimal investment, while Business and Arts and Humanities do not yet reveal opportunities that would be collaborative and thus explorative. The broad spectrum of research on *Wolbachia* underlines the multidisciplinary (Simón et al., 2017; Kaur et al., 2021; Piccinno et al., 2024) focus on this topic and its impact on various areas of science and society.

To find patterns and themes in an area, co-word analysis analyzes the keyword co-occurrence. Conceptual mapping shows the field's main subjects and trends through the networks of linked concepts. Together, they reveal the conceptual framework of the study domain. Co-word analysis uses keyword frequency and proximity to highlight the conceptual structure of a study domain and to identify major subjects and their interconnections (Pritish Baskaran et al., 2023; Tsai et al., 2020; Manjareeka, 2023; Afuye et al., 2021). WR's most common author keywords were “*Wolbachia*” (1961 occurrences) and “cytoplasmic incompatibility” (327). CI is a post-mating incompatibility that generally results in F1 inviability when infected men mate with uninfected females or females carrying a different bacterial strain than that of the male. During a previous examination of the subject, *Wolbachia*-induced CI was the sole phenomenon identified that inhibited host gene flow through a microbe–microbe interaction (Turelli and Hoffmann, 1995; Dobson et al., 1999; Adekunle et al., 2019). A recent study on several invertebrate–microbe interactions revealed that *Cardinium* symbionts, belonging to the distinct *Cytophaga–Flavobacterium–Bacteroides phylum*, can also induce CI (Martinez et al., 2022; Zhang and Lui, 2020). Numerous significant experimental and theoretical breakthroughs have persisted in substantiating the diverse roles of *Wolbachia*-induced CI in arthropod diversification. In addition to bacterial-induced CI, cytoplasmic organelles of bacterial origin, such as mitochondria and chloroplasts, can produce hybrid disorders through epistatic interactions with host nuclear genes (Hoffmann et al., 2011; Weeks et al., 2007; Hoffmann et al., 2014; Walker et al., 2011; McMeniman et al., 2009; Adekunle et al., 2019).

Studies focusing on *Drosophila* and *Wolbachia* have demonstrated the rich interactions that have been described above. The study of this aspect touches on the extent of the distribution of *Wolbachia* in *Drosophila melanogaster* groups, their reproductive activities, and the possibility of altering the characteristics of *Drosophila simulans* using various *Wolbachia* strains. Understanding the dynamics of these relationships is important. Imagine scientists removing naturally occurring *Wolbachia* from fruit flies and replacing it with one usually possessed by mosquitoes or isolating and sequencing small prokaryotic dnaA discovered within infected fruit flies to see how they impact reproduction (Solignac et al., 1994; Rousset and Stordeur, 1994; Braig et al., 1994; Bourtzis et al., 1994; Holden et al., 1993; Bressac and Rousset, 1993; Boyle et al., 1993; Rousset et al., 1992; Louis and Nigro, 1989; Binnington and Hoffmann, 1989). Understanding the concept of transfer in different *Drosophila* species and the reproductive potential of *Wolbachia* is necessary to appreciate the newly found levels of these powerful organisms that are hidden in these small animals.

Certain filarial worms have *Wolbachia*, including those that cause lymphatic filariasis. *Wolbachia*-targeting drugs, such as doxycycline and minocycline, can help reduce worm viability by impairing their reproductive ability and, as such, weaken them (Lu et al., 2012). Disease management is more effective when combined with definitive medical treatment as worms and their symbiotic bacteria are effectively addressed, resulting in improved disease management (Slatko et al., 2014; Manoj et al., 2021). Although traditional therapies such as ivermectin and albendazole have limited utility in managing patients with chronic filariasis, this prompted the investigation of residing *Wolbachia* in filarial worms (Porter and Sullivan, 2023; Simón et al., 2017; Kaur et al., 2021). Parafilarial worms are obligate and essential for *Wolbachia*, which encouraged the discovery of ways to block symbiosis to enhance treatment and minimize transmission (Bakowski and McNamara, 2019). This is particularly pertinent in areas where filariasis is an endemic. Canine heartworm disease is prevalent worldwide and is life threatening in dogs. Areas favorable for breeding mosquitoes facilitate the spread of the causative agent *D. immitis* among dogs. Concerned about the health implications of heartworms, recent veterinary researchers have made phylogenetic and biochemical observations in support of the obligatory presence of *Wolbachia* in *D. immitis*. Therefore, there is information regarding the use of antibiotics as adjunct or alternative antiparasitic therapies to manage heartworm disease in dogs. Owing to the toxicity of melarsomine adulticidal treatments, many efforts have been made to study doxycycline and other antibiotics (McHaffie, 2012).

It is obvious from the changes over time in WR thematic modeling that the research methods and/or areas of interest in the scientific circle have not remained static over time. From the archival period of to 1936–2012, other familiar topics, such as *Ae. aegypti*, mitochondrial DNA (mtDNA), parthenogenesis, and symbionts, were emphasized in the studies, which may have served to fulfill the basic biology of *Wolbachia* and its hosts, as well as their relationships. Climbing down to the period–2013-2019 some of the themes advanced to the use of endosymbionts and lymphatic filariasis, as well as *Wolbachia*, among others, and its applications. This indicates a more qualified investigation of *Wolbachia* infections, particularly regarding the control of vector-mediated diseases caused by vectors. In the present timeframe of 2020–2025, further diversification in the research path is discerned, narrowing the field to genetic diversity and onchocerciasis, and persistent attention to *Wolbachia* as an essential factor in controlling diseases carried out by *Ae. aegypti* mosquitoes. This phase marks a gradual evolution in the research trajectories, focusing on the internal complexities of *Wolbachia* in more detail in vector-borne disease control. Overall, the chronological development of WR shows progress in biologists focused initially on basic biological questions to more applied and specific studies oriented to use the symbiosis of *Wolbachia* for the control of disease-causing vectors. This shift demonstrates the active nature of science in all its facets, whereby areas of research are continually being transformed to meet new public health challenges brought about by vector-borne diseases.

Since 2015, a growing number of research topics have spanned a vast array of subjects, including periodic solutions (Fig. 9). In WR, periodic solutions are recurrent trends in mathematical models that characterize the dynamics of *Wolbachia* infections within host populations over time. Mathematical models have demonstrated efficacy in modeling and assessing mosquito-borne diseases (Hasibuan et al., 2022; Adekunle et al., 2019; Andreychuk and Yakob, 2022). In recent years, the dynamics of mosquito populations influenced by *Wolbachia* interference has attracted the attention of researchers, coinciding with technological advancements. Several mathematical models have been developed to examine the effects of the release of *Wolbachia*-infected mosquitoes on the regulation of wild mosquito populations, which can be classified into two types. Models for population replacement involved the release of both *Wolbachia*-infected males and females, whereas models for population suppression involved the release of only *Wolbachia*-infected males. The males released during population replacement or suppression may cause CI, leading to the failure of egg hatching (Hasibuan et al., 2022; Adekunle et al., 2019; Zhang and Lui, 2020; Andreychuk and Yakob, 2022; Yang et al., 2023; Guevara-Souza and Vallejo, 2015). Other emerging topics, as noted in Fig. 9, indicate growing interest within the scientific community in areas such as microbiology, genomics, disease surveillance, vector biology, and microbiota dynamics associated with arboviruses and mosquito-borne diseases, such as dengue, reflecting the evolving research landscape in these areas since 2015.

This study is limited by its exclusive reliance on the Scopus database, which potentially overlooks research from other domains, and linguistic bias, as it considers only English-language publications. Furthermore, reliance on a single data source may omit significant insights from alternative sources. Other forms of literature reviews may be included to augment the conventional analytical approach. Regularly updating the study to incorporate new articles from databases is essential, and future research may leverage various databases for more thorough analysis. Finally, the study recommends enhancing the search methodology by implementing a multimethod approach or optimizing the search strategy to achieve a deeper comprehension of research trends in the field.

## 5 Conclusions

The conclusions drawn from this study suggest that WR has experienced strong development, pushing out a fair amount of 4800 documents by the year 2024, which suggests that concern and activity of research in this field continues to increase. Citation mapping of WR shows the influence of the discipline – even though there is fluctuation in such influence as seen in 1990, where there was a peak in citation records. There have been several WR contributors, which are easy to prove because of their quantitative citation records along with publication records dating back to the late 1980s and the 1990s. This effort has not only determined the leading research activities in *Wolbachia* dissemination, but has also fostered the bonding of countries such as the USA, the UK, and China, and institutions such as CNRS and The University of Queensland, toward extend the knowledge base in this area. WR taxonomy is transdisciplinary, spanning Agricultural Sciences, Biochemistry, Genetics, Immunology and Medicine. Key themes imply that the applied aspects of research and development, particularly on a genetic basis and for disease control, are on the rise. Trends after 2015 show that the focus has shifted to microbiology and genomics, as well as disease and vector biology, with particular regard to arboviruses and mosquito-borne diseases, illustrating the changing face of the research. Essentially, all these studies can be understood as including WR as a noun, which is less static and enhances intra-, inter-, and translational collaboration fields with diverse research models that have changed over time. WR faces several important gaps, such as assessments of the long-term effects of interventions, including their ecological aspects, the integration of social sciences into the assessments of society's impact, the necessity of translating the findings into practice, and the direct targeting of disease control measures. Recommendations focus on the need for systematic efforts to develop multi-perspective thinking among experts, administering projects to assist communities in making informed decisions, outjoining practitioners in developing research agendas and methods, and implementing ‘engagement first’ approaches to *Wolbachia* studies and interventions. Overall, these deficiencies that need to be addressed, in addition to the recommendations provided, will push the WR agenda forward, thereby contributing to more effective interventions and greater acceptance of society.

## Availability of data and material

NA.

## CRediT authorship contribution statement

**Manal Mohamed Elhassan Taha:** Writing – review & editing, Writing – original draft, Visualization, Validation, Supervision, Software, Resources, Project administration, Methodology, Investigation, Funding acquisition, Formal analysis, Data curation, Conceptualization. **Siddig Ibrahim Abdelwahab:** Writing – review & editing, Writing – original draft, Visualization, Validation, Supervision, Software, Resources, Project administration, Methodology, Investigation, Funding acquisition, Formal analysis, Data curation, Conceptualization. **Hafeez Yagoub Mohamed:** Investigation, Funding acquisition, Formal analysis, Data curation. **Ahmed Jerah:** Methodology, Investigation, Funding acquisition, Formal analysis, Conceptualization. **Aied M. Alabsi:** Formal analysis, Data curation, Conceptualization. **Saleh Mohammad Abdullah:** Writing – review & editing, Visualization. **Bassem Oraibi:** Writing – review & editing, Writing – original draft, Validation. **Hassan Ahmad Alfaifi:** Writing – review & editing, Funding acquisition, Formal analysis, Data curation, Conceptualization. **Yasir Osman Hassan Babiker:** Writing – review & editing, Visualization. **Saeed Alshahrani:** Methodology, Investigation, Funding acquisition, Formal analysis, Data curation. **Abdullah Mohammed Farasani:** Writing – review & editing, Methodology. **Tawfeeq Altherwi:** Writing – review & editing, Writing – original draft, Visualization, Validation, Supervision, Software, Resources, Project administration, Methodology, Investigation, Funding acquisition, Formal analysis, Data curation, Conceptualization.

## Consent for publication

NA

## Ethics approval and consent to participate

NA.

## Funding

This research article is derived from a research grant funded by the Research, Development, and Innovation Authority (RDIA) - Kingdom of Saudi Arabia - with grant number (821-jazzan-2023-JZU-R-2-1-HW). The authors gratefully acknowledge the funding of the Deanship of Graduate Studies and Scientific Research, Jazan University, Saudi Arabia, through Project number: (JU- 20250298 -DGSSR- RP -2025).

## Declaration of competing interest

The authors declare that they have no known competing financial interests or personal relationships that could have appeared to influence the work reported in this paper.
